# Advanced therapeutic strategy for managing surgical site infections with natural nanoemulsion-antimicrobial combination

**DOI:** 10.3389/fphar.2025.1617184

**Published:** 2025-07-02

**Authors:** Fatma Alshehri, Nada K. Alharbi, AbdelNaser Zaid, Amira H. Eltrawy, Shereen Fawzy, Attia M. Gabr, Sawsan A. Zaitone, Majid Alhomrani, Abdulhakeem S. Alamri, Rasha A. Mosbah, Rana Elshimy, Abdallah Tageldein Mansour, Mahmoud M. Bendary

**Affiliations:** ^1^ Department of Biology, College of Science, Princess Nourah bint Abdulrahman University, Riyadh, Saudi Arabia; ^2^ Department of Surgery, Faculty of Medicine, Jazan University, Jazan, Saudi Arabia; ^3^ Department of General Surgery, Faculty of Medicine, Assiut University, Assiut, Egypt; ^4^ Department of Anatomy, Faculty of Medicine, University of Tabuk, Tabuk, Saudi Arabia; ^5^ Department of Medical Microbiology, Faculty of Medicine, University of Tabuk, Tabuk, Saudi Arabia; ^6^ Department of Pharmacology and Toxicology, College of Pharmacy, Qassim University, Buraidah, Saudi Arabia; ^7^ Department of Clinical Pharmacology, Faculty of Medicine Suez Canal University, Ismailia, Egypt; ^8^ Department of Pharmacology and Toxicology, Faculty of Pharmacy, University of Tabuk, Tabuk, Saudi Arabia; ^9^ Department of Pharmacology and Toxicology, Faculty of Pharmacy, Suez Canal University, Ismailia, Egypt; ^10^ Department of Clinical Laboratories Sciences, The Faculty of Applied Medical Science, Taif University, Taif, Saudi Arabia; ^11^ Research Center for Health Science, Deanship of Scientific Research, Taif University, Taif, Saudi Arabia; ^12^ Infection Control Unit, Zagazig University Hospital, Zagazig, Egypt; ^13^ Military Medical Academy, Cairo, Egypt; ^14^ Department of Microbiology and Immunology, Faculty of Pharmacy, Alhram Canadian University, Giza, Egypt; ^15^ Fish and Animal Production Department, College of Agriculture and Food Sciences, King Faisal University, Al-Ahsa, Saudi Arabia; ^16^ Department of Microbiology and Immunology, Faculty of Pharmacy, Port Said University, Port Said, Egypt

**Keywords:** SSIS, MDR, chitosan nanoemulsion, amikacin, histopathological

## Abstract

**Introduction:**

Surgical site infections (SSIs) are a significant cause of morbidity and mortality, often complicated by multidrug-resistant (MDR) pathogens and biofilm formation. This study evaluates the potential of a natural nanoemulsion containing chitosan, lavender, and curcumin, in combination with antimicrobial drugs, for treating SSIs.

**Methods:**

A comprehensive approach combining phenotypic and genotypic analyses, along with *in vitro* and *in vivo* studies, was used to assess the efficacy and safety of the combination therapy.

**Results:**

The most common SSI pathogens identified were *Staphylococcus aureus*, *Escherichia coli, Klebsiella pneumoniae, Pseudomonas aeruginosa,* and *Acinetobacter baumannii*, with 50% exhibiting MDR, biofilm formation, and multiple virulence factors. Chitosan nanoemulsion showed the lowest minimum inhibitory concentration (MIC) (300–500 μg/mL), although it exceeded the cytotoxicity safety threshold (200 μg/mL). However, it significantly enhanced the antimicrobial activity of amikacin, resensitizing resistant strains at safe concentrations. The combination therapy of amikacin and chitosan nanoemulsion demonstrated superior efficacy in reducing bacterial loads in both Gram-negative and Gram-positive infections. *In vivo* studies showed near-complete bacterial clearance by day 12. Histopathological analysis revealed enhanced wound healing, reduced inflammation, and restored tissue function. The combination of amikacin and chitosan nanoemulsion presents a promising therapeutic strategy for managing SSIs caused by MDR pathogens, improving bacterial eradication and wound healing.

**Conclusion:**

This study highlights chitosan nanoemulsion as an adjuvant therapy to combat antimicrobial resistance, enhance antibiotic efficacy, and improve SSI treatment outcomes. Further clinical studies are needed to optimize its use in patient care.

## Introduction

Surgical Site Infections (SSIs) are a major healthcare concern and a leading cause of morbidity and mortality after surgery. SSIs occur when infections develop at or near the incision site, ranging from mild, treatable cases to severe complications such as sepsis, prolonged hospitalization, or death. They significantly increase healthcare costs, extend recovery time, and often necessitate reoperation. SSIs are among the most common healthcare-associated infections (HAIs) worldwide (World Health Organization, 2018; Keely Boyle et al., 2018). These infections result from contamination by pathogenic microorganisms introduced during or after surgery. Sources include exogenous bacteria from the environment, instruments, or surgical staff, and endogenous bacteria from the patient’s skin, mucosa, or internal organs, especially with poor hygiene or compromised immunity. Common pathogens include *Staphylococcus aureus* (*S. aureus*), specially methicillin resistant *S. aureu*s (MRSA), *Escherichia coli (E. coli)*, *Enterococcus* spp., and *Pseudomonas aeruginosa (P. aeruginosa)* (Berríos-Torres et al., 2017; Keely Boyle et al., 2018). SSIs are classified by tissue depth: superficial incisional (skin or subcutaneous tissue, with redness, swelling, or discharge), deep incisional (fascial and muscle layers, with risks like abscess or necrosis), and organ/space (body cavities or organs accessed during surgery, potentially causing systemic infection or organ failure) (Horan et al., 1992).

A variety of antibiotics are used to manage SSIs, each with specific targets and mechanisms. Cefepime, a fourth-generation cephalosporin, and piperacillin/tazobactam, a penicillin–beta-lactamase inhibitor combination, act by disrupting bacterial cell wall synthesis and are effective against a broad spectrum of pathogens, including MDR strains and *P. aeruginosa* (Facciolà et al., 2019). Doxycycline, a tetracycline, inhibits protein synthesis and is effective against *S. aureus*, also offering anti-inflammatory benefits (Berríos-Torres et al., 2017). Amikacin and gentamicin, aminoglycosides targeting the 30S ribosomal subunit, are potent against Gram-negative bacteria like *P. aeruginosa* and *E. coli* but carry risks of nephrotoxicity and ototoxicity, especially with prolonged use or large treatment areas (Birgand et al., 2023). Ciprofloxacin, a fluoroquinolone, impairs DNA replication by inhibiting DNA gyrase, while imipenem, a carbapenem, also targets cell wall synthesis and is effective against MDR organisms (Berríos-Torres et al., 2017; Birgand et al., 2023). Trimethoprim/sulfamethoxazole, which inhibits folate synthesis, is commonly used against *Staphylococcus* spp. and certain Gram-negatives (Birgand et al., 2023). Despite their efficacy, many antibiotics face reduced effectiveness due to resistance mechanisms, especially in *P. aeruginosa* and *S. aureus* (Berríos-Torres et al., 2017). This highlights the need for safer, more effective strategies, such as combination therapies, to overcome resistance and minimize adverse effects.

Preventing SSIs requires a full perioperative approach: preoperative antibiotic prophylaxis, proper skin preparation, and patient optimization; intraoperative aseptic technique, reduced surgery time, and sterile tools; postoperative wound care, early infection detection, timely antibiotics, and patient education (National Institute for Health and Care Excellence, 2020; Horan et al., 1992). SSIs remain a critical challenge affecting patient outcomes and healthcare systems. Understanding risk factors and preventive strategies is essential to reduce their occurrence and improve patient safety. Despite advances, ongoing optimization of surgical techniques, patient care, and prevention protocols is needed (Birgand et al., 2023; Uçkay et al., 2013). Managing SSIs with natural products has gained interest as an alternative or adjunct to antibiotics due to their antimicrobial, anti-inflammatory, and wound-healing effects. Compounds such as tea tree oil, garlic, aloe vera, lavender oil, curcumin, chitosan, manuka honey, echinacea, calendula, and neem show promise in preventing infections, promoting tissue regeneration, and supporting faster recovery (Sharifi-Rad et al., 2018; Mori et al., 2016). Lavender oil, curcumin, and chitosan offer distinct advantages in SSI management. Lavender oil provides broad-spectrum antimicrobial action against common SSI pathogens and reduces pain, inflammation, and anxiety, making it more gentle and less irritating for prolonged use (Mori et al., 2016). Curcumin’s potent anti-inflammatory and antioxidant properties promote tissue repair and modulate immune responses, outperforming other anti-inflammatory agents like garlic or ginger, with a low toxicity profile allowing safe long-term use (Priyadarsini, 2014; Mosallam et al., 2023). Chitosan combines antimicrobial effects with wound protection by forming a barrier that prevents bacterial invasion and supports cell regeneration, surpassing polysaccharides like alginate in sustaining active compound release and enhancing healing (Matica et al., 2019). Together, these natural products provide multifaceted benefits, making them more effective and safer than other compounds in managing SSIs.

Of not, the presence of multi-virulent and multidrug-resistant (MDR) pathogens complicates both infection progression and treatment outcomes (Abd El-Hamid et al., 2023; Elsayed, et al., 2022). These pathogens not only exhibit resistance to multiple antibiotic classes but also possess enhanced virulence traits such as toxin production, immune evasion mechanisms, and biofilm formation, which further protect them from host defenses and therapeutic agents. As a result, managing infections caused by such organisms has become increasingly difficult, particularly in chronic and surgical wound settings. In this context, combination therapies—integrating antimicrobial agents with bioactive compounds or delivery systems—have emerged as a promising strategy (Ghaly et al., 2023; Mosallam et al., 2021). We hypothesize that combining antimicrobials with the selected nanoemulsions will enhance antimicrobial, anti-inflammatory, and wound-healing effects in SSI management over monotherapy. These nanoemulsions are expected to improve drug bioavailability and therapeutic efficacy, resulting in better infection control, reduced inflammation, and faster tissue regeneration. This combination aims to achieve superior outcomes, including lower infection rates, quicker healing, and fewer side effects, offering a more effective and sustainable treatment strategy for SSIs. The study will evaluate and identify the most effective combination through *in vitro* and *in vivo* studies focused on antimicrobial activity, anti-inflammatory effects, and wound healing. This study will assess how these nanoemulsions enhance antimicrobial performance and whether they improve infection control, accelerate tissue repair, and shorten healing time compared to antimicrobial treatment alone. Additionally, the research will investigate their potential to reduce side effects such as antibiotic resistance and toxicity. By identifying the optimal formulation, the study aims to develop novel, sustainable treatments that improve patient outcomes and reduce healthcare burdens in SSI management.

## Materials and methods

### Characterization of pathogenic bacteria in surgical site infections following abdominal surgery

#### Phenotypic identification

A total of 150 wound swab samples were obtained from surgical sites following abdominal surgeries across various hospitals in Egypt. The infected areas were first cleaned, and then cotton sterile swabs were used to collect the clinical samples, which were placed into brain heart infusion broth to allow for pathogen enrichment. These samples were incubated for 24 h at 37°C to promote bacterial growth. Afterward, a sample from the enriched culture was spread onto nutrient agar plates. The isolated colonies were identified through standard microbiological techniques, including assessment of cultural and biochemical properties, in addition to Gram staining (Collee et al., 1996; Madigan et al., 2018). Positive growth was recorded when the clinical samples exhibited a colony count greater than 10^6^ CFU/mL. This threshold is commonly used to differentiate between contamination and actual infection. On the other hand, higher colony counts were used to select the pathogen when mixed isolates were detected (Lora et al., 2027).

#### Molecular confirmation

Sequencing of the species-specific 16S rRNA gene was employed to accurately identify the species and provide detailed information on their genetic composition and resistance characteristics. Bacterial DNA was extracted from according to the provided protocol. The 16S rRNA gene was amplified using the primers F: GGT​TAC​CTT​GTT​ACG​ACT​T and R: AGA​GTT​TGA​TCC​TGG​CTC​AG (Weisburg et al., 1991). PCR amplification started with denaturation for 10 min at 94°C, followed by 35 cycles of denaturation for 30 s at 94°C, annealing for 1 min at 56°C, and extension for 1 min at 72°C, with a final extension for 10 min at 72°C. The PCR products were analyzed using 1% agarose gel electrophoresis and visualized under UV light after staining with ethidium bromide (Sambrook and Russell, 2001). The purified amplicons were then sequenced using the ABI 3730xl DNA sequencer at GATC Biotech AG, and the sequences were compared to NCBI databases for species identification.

#### Antimicrobial sensitivity patterns

Both the disk diffusion method (Kirby-Bauer) and the microdilution method for determining the minimal inhibitory concentration (MIC) were employed to evaluate antimicrobial resistance patterns in triplicate (Lewis et al., 2025). The antimicrobial susceptibility of the bacterial isolates was assessed by testing their resistance or sensitivity to the empirical antimicrobial drugs which commonly prescribed, both in disc and powder form, sourced from Sigma-Aldrich. The most commonly prescribed antibiotics for preventing SSI were evaluated, including Cefepime (FEP, 30 µg), Doxycycline (DOX, 30 µg), Amikacin (AK, 30 µg), Piperacillin/Tazobactam (TZP, 100/10 µg), Ciprofloxacin (CIP, 5 µg), Imipenem (IMP, 10 µg), Gentamicin (CN, 10 µg), and Trimethoprim/Sulfamethoxazole (SXT, 1.25/23.75 µg). The resistance or susceptibility patterns were assessed (Lewis et al., 2025; European Committee on Antimicrobial Susceptibility Testing, 2028). The multi-drug-resistant (MDR) isolates, exhibiting resistance to one or more antimicrobial agents from at least three different categories (Magiorakos et al., 2012), were selected for further analysis in this study. The antimicrobial resistance power was evaluated by calculating the Multiple Antibiotic Resistance (MAR) index. The MAR index was determined by comparing the number of antimicrobials the isolate was resistant to the total number of antimicrobials tested. A higher MAR index reflects increased antimicrobial exposure, commonly associated with high-risk environments, and offers important insights for shaping infection control and antimicrobial stewardship strategies (Mir et al., 2022).

#### Detection of biofilm production

Biofilm production was assessed for the detected MDR isolates using the microtiter plate assay. Overnight bacterial cultures in Luria-Bertani (LB) or Tryptic Soy Broth (TSB) were diluted to an OD of 0.1 at 600 nm and 200 µL of the suspension was added to each well of a 96-well polystyrene plate. Wells containing sterile broth served as negative controls; however, positive controls were included using a known biofilm-producing strain (*P. aeruginosa* ATCC 27853), and all tests were performed in triplicate. Plates were incubated statically at 37°C for 24 h, then washed three times with phosphate-buffered saline (PBS) to remove non-adherent cells. Biofilms were stained with 0.1% crystal violet for 15 min, washed, and air-dried. The bound stain was solubilized with 95% ethanol, and absorbance was measured at 570 nm using a microplate reader. Biofilm production was detected based on OD values relative to negative control as non-producers (Stepanović et al., 2007).

### Molecular detection of virulence genes of multidrug-resistant biofilm-producing isolates

The virulence gene profiles of all multidrug-resistant (MDR) biofilm-producing *S. aureus* isolates were determined by detecting the genes *sea-sed, eta, etb, tst, and pvl* (Mehrotra et al., 2000). Additionally, the virulence genes of MDR biofilm-producing *E. coli*, *P. aeruginosa*, *K. pneumoniae* and *Acinetobacter baumannii* (*A. baumannii*) were identified. In *E. coli*, the genes *ompA, kpsMTII, hly, astA, fimH,* and *vt2e* were detected (Nataro and Kaper, 1998; Kaper et al., 2004). In *P. aeruginosa*, the genes *aprA, lasB, pIcH,* and *toxA* were identified (Nikbin et al., 2012; Wiegand et al., 2007). In *A. baumannii*, the virulence genes *bap, fimH, csgA,* and *csuE* were detected (Tomaras et al., 2003; Abdi et al., 2020; Thummeepak et al., 2016). For *K. pneumoniae*, PCR detection of *rmpA*, *uge*, *fimH1*, and *wabG* was performed using species-specific primers and amplification conditions as described in the studies by (Siu et al., 2011; Alcántar-Curiel and Girón, 2015; El Fertas-Aissani et al., 2013), with DNA extracted from clinical isolates serving as the template for gene amplification. Positive and negative controls were included in all PCR experiments. Notably, all runs adhered to PCR unidirectional workflow guidelines. The amplified products were resolved on a 2% agarose gel stained with ethidium bromide (0.5 μg/mL) and subjected to electrophoresis at 70 V for 45 min. The gel was subsequently visualized using a UV illuminator for photo documentation, and the results were analyzed. The molecular weight of each amplified product was determined using a 100 bp DNA ladder (Thermo Scientific, Molecular Biology).

### Formulation and characterization of nanoemulsions containing curcumin, lavender oil, and chitosan

Curcumin, lavender oil, and chitosan nanoemulsions were synthesized and validated by the Egyptian Atomic Energy Authority (EAEA) before being provided for use in the study. Curcumin, lavender oil, and chitosan were purchased from Sigma-Aldrich, a leading supplier of laboratory-grade chemicals and materials, to be used in the development of nanoemulsions. These natural compounds were chosen due to their known therapeutic properties, including anti-inflammatory, antimicrobial, and antioxidant effects. The nanoemulsion production and validation processes were carried out in the Egyptian Atomic Energy Authority (EAEA), a prominent governmental research institution in Egypt.

### Assessment of the antimicrobial activity of natural nanoemulsions using the broth microdilution technique

The antimicrobial activities of natural nanoemulsion were evaluated in triplicates using the broth microdilution method, as described by Clinical and Laboratory Standards Institute (CLSI) guidelines (Lewis et al., 2025). Briefly, the natural nanoemulsion was prepared by dissolving the emulsified compounds in DMSO, followed by the formulation of different concentrations ranging from 0.1 to 1,000 μg/mL. Bacterial isolates were cultured overnight in nutrient broth, and the bacterial suspension was adjusted to a concentration of approximately 10^6^ CFU/mL. The microdilution assay was performed in a 96-well microtiter plate by adding 100 µL of bacterial suspension to 100 µL of each concentration of the nanoemulsion. The plate was incubated at 37°C for 18–24 h. After incubation, the bacterial growth was determined by measuring the absorbance at 600 nm using a microplate reader. The MIC was defined as the lowest concentration of nanoemulsion that inhibited visible bacterial growth. For control, positive and negative wells containing bacterial culture with solvent and without the nanoemulsion, respectively, were included in each assay (Dos-Santos et al., 2025). The antibacterial activity was further confirmed by observing the visual turbidity reduction, which indicated antimicrobial efficacy.

### Investigation of changes in antimicrobial sensitivity patterns of tested drugs upon treatment with Sub-MIC of a natural nanoemulsion

The microdilution method was used in triplicate to investigate changes in antimicrobial sensitivity patterns. Bacterial strains were cultured to standardized inoculum densities (e.g., 10^6^ CFU/mL) in broth media. Serial two-fold dilutions of the tested antimicrobial drugs were prepared in 96-well microtiter plates. Sub-MIC level of each natural nanoemulsion was added to the wells containing the drugs, followed by incubation under optimal conditions for 16–20 h. Bacterial growth was assessed by measuring optical density (OD) or visual inspection, and the minimum inhibitory concentrations (MICs) were detected to evaluate the impact of nanoemulsion. After that the changes in the resistance patterns were recorded.

### Assessment of the interaction between natural nanoemulsion and the commonly used antimicrobial drugs using the checkerboard method

The antimicrobial synergy between natural nanoemulsion and antimicrobial drug was evaluated using the checkerboard method, as described by Odds (2003). Briefly, bacterial isolates were cultured overnight and adjusted to an OD of 0.1 at 600 nm, corresponding to approximately 10^6^ CFU/mL. A 96-well microtiter plate was prepared, and serial two-fold dilutions of both the natural nanoemulsion and the previous evaluated antimicrobial drugs were made along the horizontal and vertical axes of the plate, respectively. The final concentration of nanoemulsion ranged from 0.5 to 128 μg/mL, and the final concentration of previous evaluated antimicrobial drugs ranged from 0.5 to 64 μg/mL. The bacterial suspension was added to each well, and the plate was incubated at 37°C for 18–24 h. After incubation, the MIC (minimum inhibitory concentration) of each compound alone and in combination was determined by visual inspection of bacterial growth and by measuring the absorbance at 600 nm. The interaction between the two agents was assessed using the fractional inhibitory concentration (FIC) index, calculated as follows: FIC index = (MIC of plant extract when combined/MIC of plant extract alone) + (MIC of antimicrobial drug when combined/MIC of antimicrobial drug alone). A FICI value of ≤0.5 indicates synergy, >0.5 to 1 indicates additive effect, >1 to 4 indicates indifference, and >4.0 indicates antagonism (Odds, 2003; Tängdén, 2014). The results were interpreted to determine the potential synergistic, additive, or antagonistic effects between the natural nanoemulsion and antimicrobial drug.

### *In vitro* cytotoxicity testing

To assess the safety concentration of the most effective nanoemulsion, its cytotoxic effects were evaluated on fibroblast. Cells were cultured in Dulbecco’s Modified Eagle Medium (DMEM) supplemented with 10% fetal bovine serum (FBS) and 1% penicillin-streptomycin, maintained at 37°C in a 5% CO_2_ humidified atmosphere. Cells were seeded at a density of 5 × 10^4^ cells per well in a 96-well plate and incubated overnight for adherence. The most effective nanoemulsion at the designated concentrations was applied to the wells, and cytotoxicity was assessed using the MTT assay after 24 and 48 h of exposure (Arora et al., 2009). The percentage of viable cells was determined by measuring absorbance at 570 nm using a microplate reader. The results were analyzed to determine the cytotoxic threshold and optimal safe concentration for further *in vivo* application.

### *In vivo* study and histopathological examination

#### Animals

A total of ninety (90) healthy 9-week-old male Albino rats were utilized in this study. All animals were individually housed under controlled environmental conditions, maintaining a constant temperature of 20°C ± 2°C with a 12-hour light/dark cycle. They had unrestricted access to food and water. To promote natural behaviors, the cages were enriched with carton houses, wooden boards, small gnawing blocks, and wood wool for nesting before and throughout the experiment.

### Selection of challenge pathogens, antimicrobial agent, and nanoemulsion formulation

The study selected one Gram-positive and one Gram-negative pathogen as challenge organisms, both of which must exhibit MDR patterns, high virulence, and biofilm formation. These pathogens should harbor key virulence genes essential for invasion and immune evasion, making them ideal candidates for evaluating therapeutic efficacy. The selection of the antimicrobial agent for the *in vivo* test was based on its broad-spectrum activity against MDR pathogens, its ability to overcome bacterial resistance mechanisms, and its effectiveness in targeting biofilm-producing bacteria. Furthermore, its strong synergistic effect in resensitizing resistant strains enhances its potential for combination therapy, making it a promising candidate for improving antimicrobial efficacy while mitigating resistance development. Additionally, the most effective nanoemulsion, characterized by the lowest MIC and a significant ability to enhance the antimicrobial efficacy of selected drugs, was chosen for use in the study.

### *In vivo* evaluation of the wound healing effect

#### Animal acclimation and grouping

The animals were randomly assigned to nine experimental groups (n = 10 per group): GP1 (non-infected, non-treated control group where placebo (normal saline) was applied as a negative control), GP2 (Gram-negative-infected, non-treated group, where placebo (normal saline) was applied instead of a drug), GP3 (Gram-negative -infected group treated with the most effective nanoemulsion), GP4 (Gram-negative -infected group treated with selected antimicrobial), GP5 (Gram-negative -infected group treated with a combination of selected antimicrobial and the most effective nanoemulsion), GP6 (Gram-positive -infected, non-treated group, where placebo (normal saline) was applied instead of a drug), GP7 (Gram-positive -infected group treated with the most effective nanoemulsion), GP8 (Gram-positive -infected group treated with selected antimicrobial), and GP9 (Gram-positive -infected group treated with a combination of selected antimicrobial and the most effective nanoemulsion).

#### Wound induction

To induce wounds, the animals were anesthetized via intraperitoneal injection of a ketamine-xylazine (K–X) cocktail, consisting of ketamine (100 mg/kg) and xylazine (10 mg/kg) (Van Pelt, 1977). The exposed shaved skin was sterilized using 70% ethanol, and full-thickness wounds (5 mm in diameter) were created along the dorsal midline. The wounds remained open without dressing for the study duration.

#### Wound contamination and infection confirmation

The selected clinical isolates were cultured in Mueller-Hinton broth (MHB) until reaching the logarithmic growth phase. The bacterial suspension was then centrifuged (1,000 g for 15 min), the supernatant was discarded, and the pellet was resuspended in sterile phosphate-buffered saline (PBS) at ∼10^9^ CFU/mL. Immediately after wound creation, 25 μL of bacterial suspension was applied to each wound site. The establishment of active infection was confirmed using standard culturing techniques over the subsequent 3 days post-contamination (Håkansson et al., 2019).

#### Treatment application and healing assessment

Topical treatments were applied once daily for 12 consecutive days using a Carbopol hydrogel as the delivery carrier. One group received 10 μL of the selected antimicrobial agent at 5 mg/mL, chosen based on its moderate resistance profiles. Another group received the same antimicrobial dose combined with the most effective nanoemulsion at a determined safe concentration. The wound healing process, as determined by the granulation and inflammation scores (Table 1), along with histopathological examination, was assessed through gross examination using digital imaging and microbiological analysis via specific plate counts at 0, 4, 8, and 12 days post-treatment.

**TABLE 1:** *In Vivo* granulation and inflammation score criteria.

Score value	Granulation tissue criteria (*In vivo*)	Inflammation criteria (*In vivo*)
1	No granulation tissue	No inflammation
2	Mild granulation tissue: Thin layer with fragile blood vessels, red to pink color, minimal texture, small tissue formation covering <20% of wound	Mild inflammation: Slight swelling and redness, minimal increase in blood flow, no pus or exudate, mild tenderness
3	Moderate granulation tissue: Denser layer, prominent blood vessels, pink to reddish color, rough texture, moderate tissue formation covering 20%–50% of wound	Moderate inflammation: Noticeable swelling and redness, moderate increase in blood flow, slight exudate, some pain or tenderness
4	Full mature granulation tissue: Well-formed, thick tissue, fully developed blood vessels, firm consistency, pink/pale color, significant tissue growth covering >50% of wound	Severe inflammation: Intense swelling, redness, heat, significant blood flow, visible vessels, pus or exudate, significant pain or tenderness, possible ulceration

#### Bacterial bioburden determination

To quantify bacterial load, tissue specimens were individually weighed and homogenized in 2 mL of PBS. The resulting homogenates and collected wound exudates were serially diluted (1:10, 1:100, 1:1,000, 1:10,000) and plated on Cetrimide Agar and Mannitol Salt Agar in triplicate. Plates were incubated for 18 h at 37°C under a humidified atmosphere. The bacterial load was expressed as log10 CFU/mL (Georgiou et al., 2020).

#### Histopathological evaluation

Mice were euthanized at predetermined time points post-wounding following an ethically approved animal protocol. Formalin-fixed skin specimens underwent conventional processing, involving sequential dehydration with ethanol (70%, 80%, 90%, and 100%) followed by purification with xylene. The samples were embedded in paraffin wax, sectioned into 4.5 μm-thick slices, and stained using Hematoxylin and Eosin (H&E). Tissue sections were examined under a light microscope (Olympus BX43), and histological images were captured using an Olympus DP21 camera with CellSens Dimension software (Bancroft and Gamble, 2007). Wound healing was scored based on a five-point scale, evaluating re-epithelialization, inflammation, granulation tissue formation, and collagen deposition (van de Vyver et al., 2021). The median score was derived from at least 35 microscopic fields (5 fields per section across 7 sections per group).

#### Biochemical analysis of serum by ELISA

Serum levels of glutathione (GSH), transforming growth factor-beta 1 (TGF-β1), interleukin-10 (IL-10), tissue inhibitor of metalloproteinases (TIMP), and matrix metalloproteinase-9 (MMP-9) were quantified using commercially available ELISA assays, strictly adhering to the manufacturer’s instructions (Thermo Fisher Scientific, USA).

### Statistical analysis

Heatmaps were generated using the R programming language (version 4.2.2) to visually illustrate the variation in bacterial load, cytokines level, antimicrobial resistance and treatment response across groups and time points. Additionally, The Friedman test was used to assess changes in bacterial load over time within treatment groups (GP2–GP9). The Kruskal-Walli’s test was applied to compare bacterial loads across groups at each time point (Days 0, 4, 8, and 12). For comparing two specific groups (e.g., GP2 vs. GP3), the Mann-Whitney U test was used. Pairwise comparisons of antimicrobial effects between Curcumin, Lavender, and Chitosan across various bacteria were based on reported p-values, with significance thresholds set at *p* < 0.05 (*) and *p* < 0.01 (**). Analyses focused on identifying significant differences in bacterial load and treatment efficacy.

## Results

### Phenotypic characterization of bacterial isolates in surgical site infections

In our study, we identified several pathogens responsible for SSIs through standard microbiological techniques, which included the examination of culture characteristics, Gram staining, and various biochemical tests. Out of 150 wound swab samples, 98 exhibited positive growth with a uniform colony count exceeding 10^6^ CFU/mL, resulting in a prevalence rate of 65.3%. Growth with a colony count below 10^6^ CFU/mL was disregarded. Bacterial isolates were identified using standard microbiological methods, yielding the following prevalence rates: 30% (26/98) for *S. aureus*, 23.5% (23/98) for *E. coli*, 16.3% (16/98) for *Klebsiella pneumoniae (K. pneumoniae)*, 20.4% (20/98) for *P. aeruginosa*, and 13.3% (13/98) for *A. baumannii.* The DNA sequences obtained from PCR of 16S RrNA genes were compared with published sequences using the BLAST tool (http://www.ncbi.nlm.nih.gov/blast). The purified PCR products were analyzed to determine its similarity to sequences available in GenBank. Sequence alignment showed over 96% nucleotide identity between the sequenced genes of the bacterial strain used in this study and previously published data in GenBank. Molecular identification results aligned with phenotypic identification, confirming the detected isolates as *S. aureus*, *E. coli, K. pneumoniae, P. aeruginosa*, and *A. baumannii*, with accession numbers PQ892234- PQ892258, PQ885533-PQ885555, PQ885572-PQ885587, PQ892259-PQ892277, PQ885600-PQ885612 respectively.

### Investigation of antimicrobial resistance patterns, MAR indices, and biofilm production in the detected clinical bacterial isolates

**FIGURE 1:**
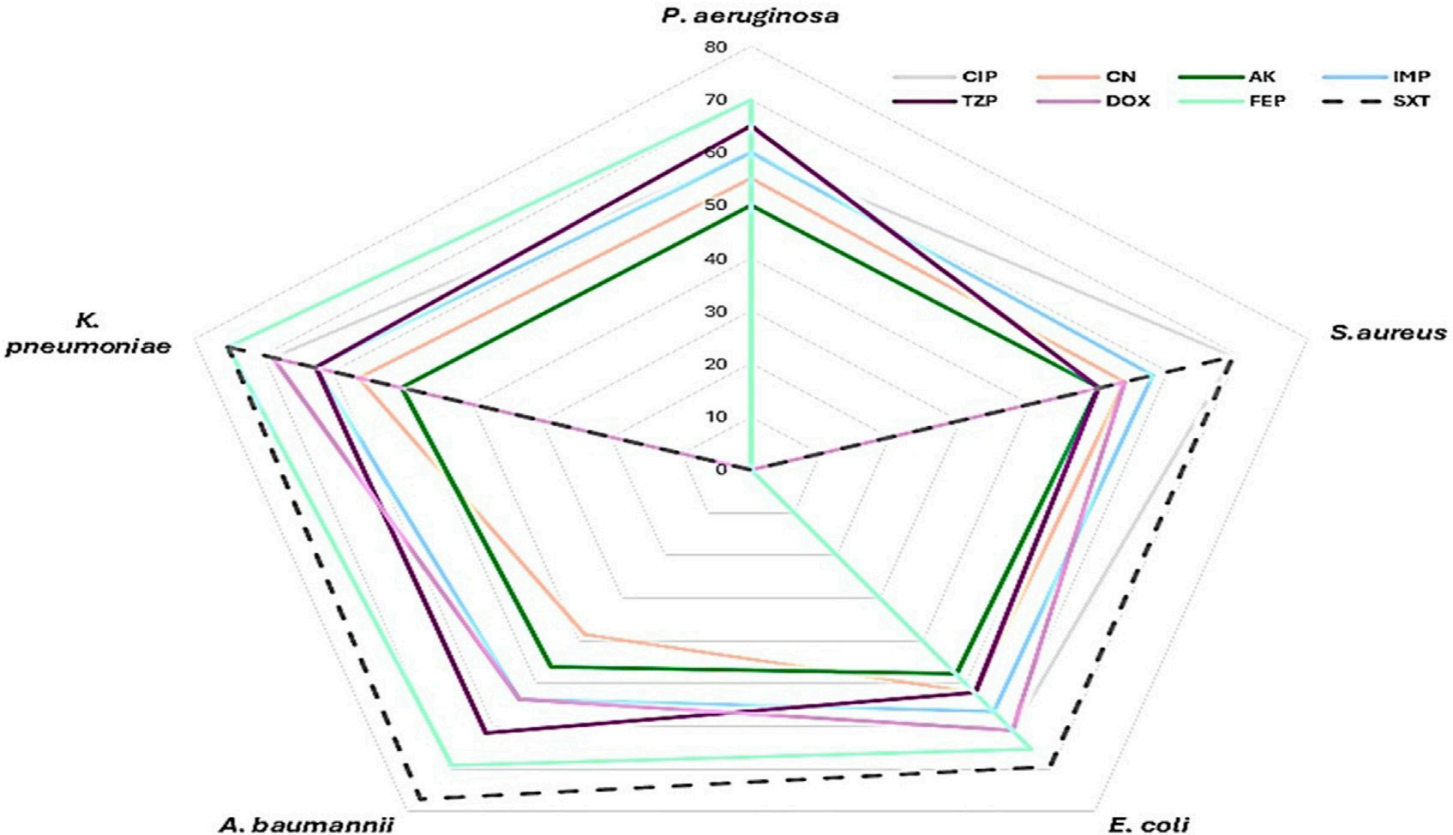
Antimicrobial Resistance Profile of Pathogens in Surgical Site Infections (SSI). This radar chart presents the resistance levels of the detected pathogens (*P. aeruginosa, K. pneumoniae, S. aureus, A. baumannii, E. coli*) against multiple antimicrobial agents. The antimicrobial agents tested include ciprofloxacin (CIP), gentamicin (CN), amikacin (AK), imipenem (IMP), ceftazidime (FEP), doxycycline (DOX), piperacillin-tazobactam (TZP), and trimethoprim-sulfamethoxazole (SXT). Resistance levels are plotted on a scale from 0% to 80%, with each antimicrobial agent represented by a distinct color as shown in the figure legend.

The antimicrobial sensitivity patterns of bacterial isolates from SSIs showed varying resistance and susceptibility across species and drugs (Figure 1). Overall, aminoglycoside such as gentamicin and amikacin in addition to imipenem appeared to be the most effective antimicrobial drugs, while sulfamethoxazole-trimethoprim and cefepime were the least effective across multiple bacterial isolates. *A. baumannii* and *K. pneumoniae* exhibited the highest resistance levels. *P. aeruginosa* and *E. coli* displayed variable sensitivity patterns, with some antimicrobial drugs maintaining efficacy, whereas *S. aureus* remained moderately sensitive to several antimicrobial drugs, including ciprofloxacin and amikacin. The MAR index revealed a high prevalence of multidrug resistance (MAR ≥0.5) among the isolates: 85% of *P. aeruginosa*, 69.2% of *S. aureus*, 73.9% of *E. coli*, 69.2% of *A. baumannii*, and 81.3% of *K. pneumoniae* exhibited MAR indices above 0.5. Additionally, MDR combined with biofilm production was detected in 60% of *P. aeruginosa*, 42.3% of *S. aureus*, 47.8% of *E. coli*, 61.5% of *A. baumannii*, and 68.7% of *K. pneumoniae*. Overall, 52 isolates (53%) demonstrated both MDR and biofilm-forming ability.

### Virulence profiles of MDR biofilm producing isolates

Virulence gene distribution varied across species. In *P. aeruginosa*, *toxA* was most prevalent (66.7%), followed by *aprA* and *lasB* (50% each), while *pIcH* was least common (41.7%). In *S. aureus*, the most frequently detected genes were *eta* and *tst* (54.5%), followed by sec (45.5%), and *see*, *etb*, and *luk-pvl* (36.4%). The least common were *sea*, *seb*, and *sed*, each detected in 27.3% of isolates. In *E. coli*, *hly* dominated (81.8%), followed by *astA* and *fimH* (63.6%), *kpsMTII* (54.5%), and *ompA* and *vt2e* (27.3%). *A. baumannii* showed high rates of *csgA* (85.7%) and *csuE* (71.4%), with *fimH* and bap each in 42.9%. In *K. pneumoniae*, *rmpA* and fimH1 were most common (63.6%), followed by *wabG* (54.5%) and *uge* (45.5%). Multivirulence, defined as presence of ≥3 virulence genes, was significantly associated with MDR and biofilm production. Of 52 MDR biofilm-forming isolates, 26 (50%) were multivirulent: 41.7% in *P. aeruginosa*, 54.5% in *S. aureus* and *E. coli*, 57.1% in *A. baumannii*, and 45.5% in *K. pneumoniae*.

### Antimicrobial effects of natural nanoemulsions

**FIGURE 2:**
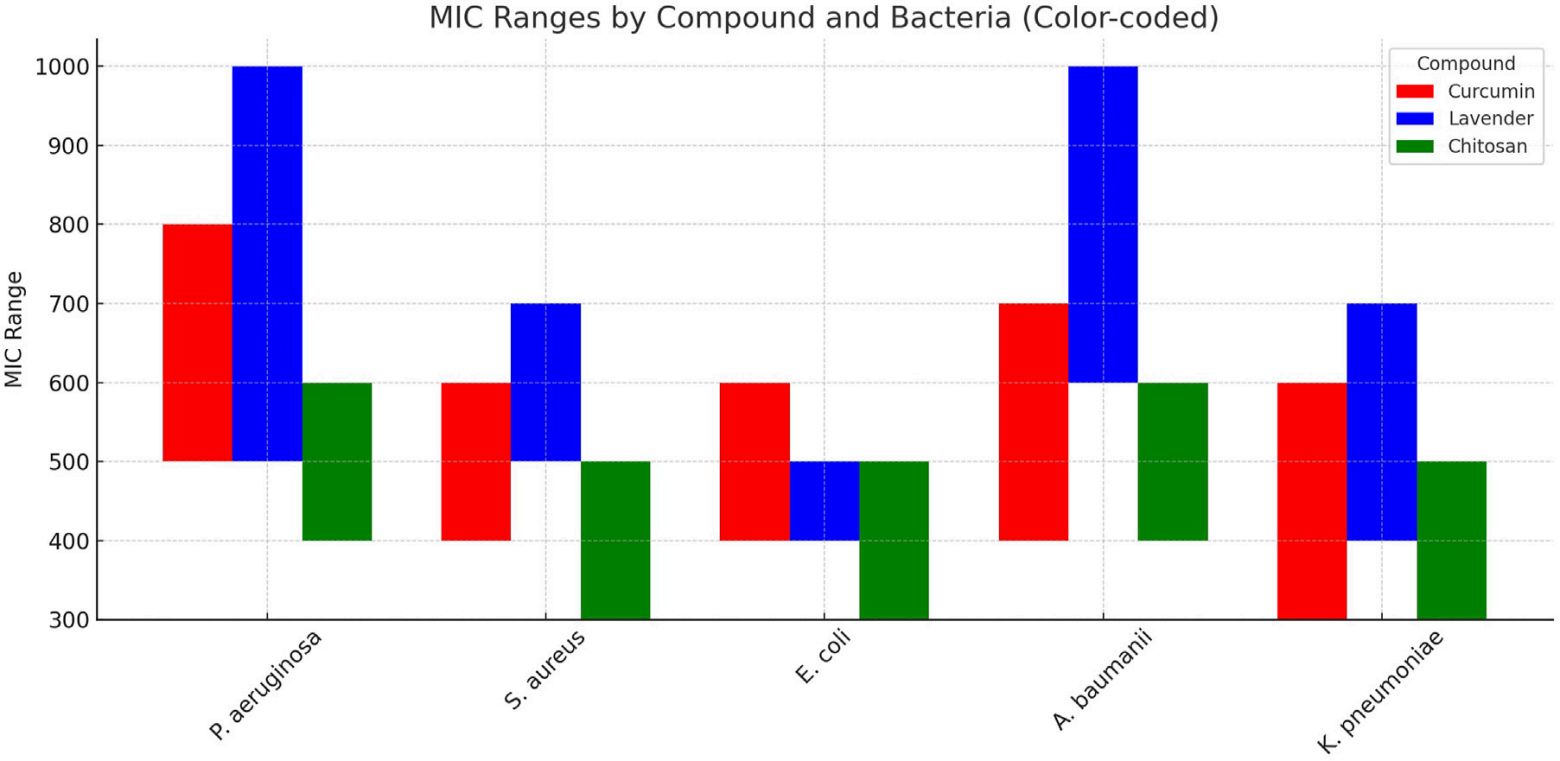
Minimum Inhibitory Concentration (MIC) ranges of curcumin, lavender, and chitosan nanoemulsions against multi-virulent, MDR, biofilm-producing clinical isolates*.* This bar chart illustrates the comparative MIC ranges (µg/mL) of three natural nanoformulations—curcumin (red), lavender (blue), and chitosan (green)—tested against *P. aeruginosa*, *MRSA*, *E. coli*, *A. baumannii*, and *K. pneumoniae*.

The antimicrobial activity of natural nanoemulsions was evaluated against 26 multi-virulent, multidrug-resistant (MDR), biofilm-producing clinical isolates, including *P. aeruginosa* (5 isolates), *S. aureus* (6), *K*. *pneumoniae* (5), *A*. *baumannii* (4), and *E*. *coli* (6). The tested nanoemulsions contained chitosan, curcumin, and lavender. The MIC results showed varying levels of antimicrobial activity among the nanoformulations, with chitosan nanoemulsion consistently exhibiting the lowest MIC values across all tested bacterial species, highlighting its superior antimicrobial efficacy (Figure 2). For *P. aeruginosa*, the MIC of chitosan ranged from 400 to 600 μg/mL, which was lower than curcumin (500–800 μg/mL) and lavender (500–1,000 μg/mL). In *S. aureus*, chitosan exhibited an MIC of 300–500 μg/mL, compared to 400–600 μg/mL for curcumin and 500–700 μg/mL for lavender. *E. coli* showed a similar trend, with chitosan MIC values of 300–500 μg/mL, while curcumin and lavender recorded MICs of 400–600 μg/mL and 400–500 μg/mL, respectively. Against *A. baumannii*, chitosan nanoemulsion again showed lower MIC values (400–600 μg/mL) compared to curcumin (400–700 μg/mL) and lavender (600–1,000 μg/mL). In *K. pneumoniae*, chitosan also achieved the lowest MIC range (300–500 μg/mL), outperforming curcumin (300–600 μg/mL) and lavender (400–700 μg/mL).

Statistical analysis supported these findings, revealing significant differences in antimicrobial activity for chitosan nanoemulsion in specific comparisons. In *P. aeruginosa*, chitosan was significantly more effective than curcumin (*p-values* = 0.04). Similarly, in *S. aureus* and *A. baumannii*, chitosan showed significantly higher antimicrobial activity compared to lavender, with *p-values* of 0.02 in both cases. These results highlighted chitosan nanoemulsion as the most effective agent among the tested natural nanoformulations, particularly against *P. aeruginosa*, *S. aureus*, and *A. baumannii*.

### Antimicrobial drug combinations with nanoemulsions

#### Combination of antimicrobial drugs with chitosan nanoemulsion

**FIGURE 3:**
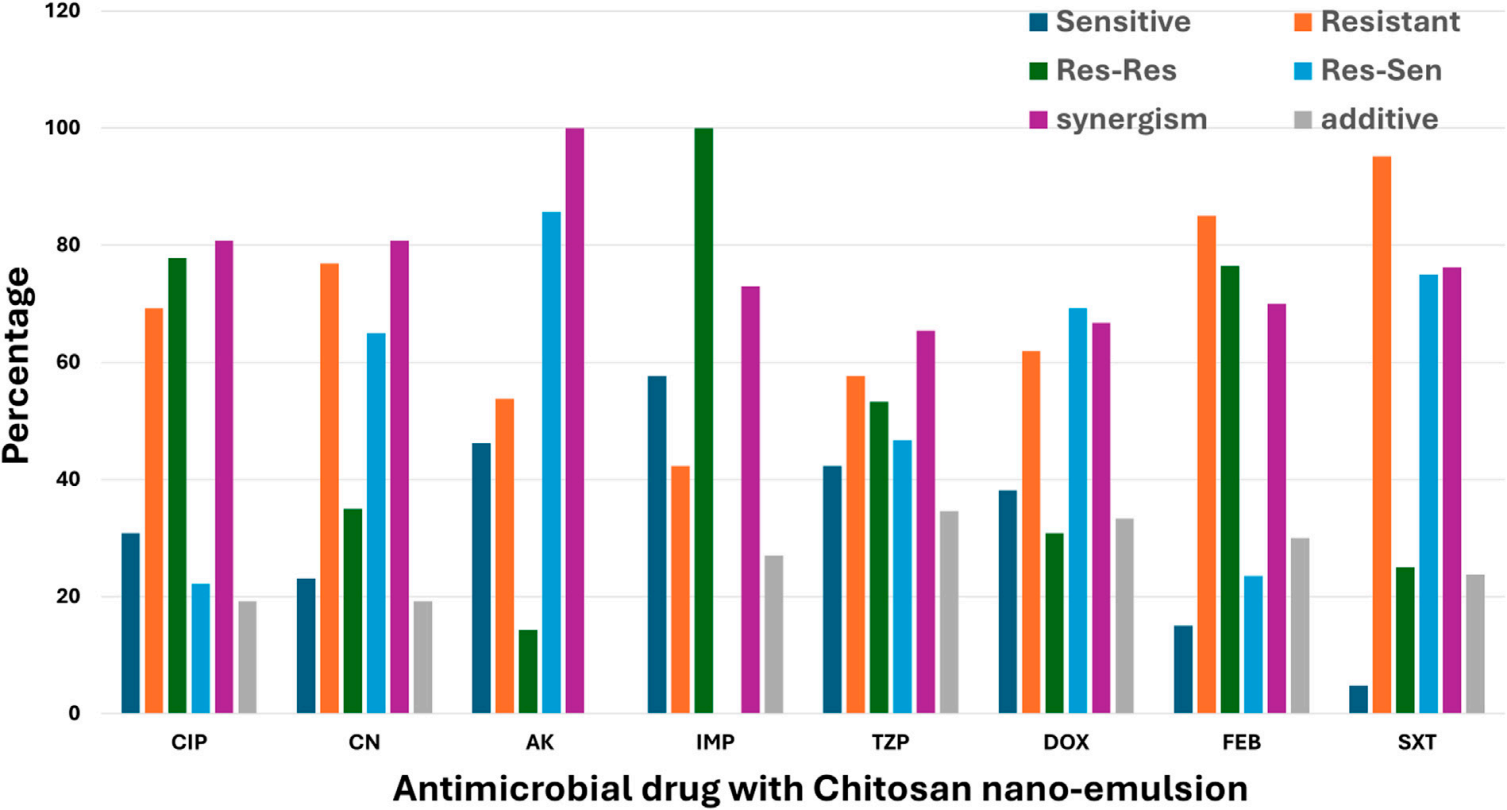
Effect of Chitosan Nanoemulsion on Antimicrobial Resistance Patterns of Pathogens in Surgical Site Infections (SSI). This bar chart illustrates the impact of chitosan nanoemulsion on the antimicrobial resistance profiles of various pathogens. The categories presented are: Sensitive (Sen), where the pathogen is susceptible; Resistant (Res), where the pathogen remains resistant; Res-Sen, where initially resistant pathogens become sensitive following treatment with chitosan nanoemulsion; and Res-Res, where resistant pathogens remain unaffected. The data are displayed as percentages for each category, demonstrating the modulation of resistance patterns by chitosan nanoemulsion.

The resensitization potential of chitosan nanoemulsion Was inconsistent across different antimicrobials (Figure 3). The highest resensitization rate was observed with amikacin at 85.7%, indicating a strong reversal of resistance in previously resistant isolates. This was followed by sulfamethoxazole-trimethoprim at 75%, doxycycline at 69.2%, and gentamicin at 65%. Moderate resensitization was noted with piperacillin-tazobactam at 46.7%, while lower rates were recorded for ciprofloxacin at 22.2% and cefepime at 23.5%. Notably, no resensitization was observed with imipenem, suggesting that chitosan nanoemulsion had no effect in restoring susceptibility to this antibiotic. The combination of chitosan nanoemulsion with conventional antimicrobials resulted in high rates of synergism (FIC <0.5), particularly with amikacin, showing a synergistic effect in 100% of tested isolates. This was followed by ciprofloxacin and gentamicin, both at 80.8%, and sulfamethoxazole-trimethoprim at 76.2%. Moderate synergism was observed with imipenem at 73%, cefepime at 70%, doxycycline at 66.7%, and piperacillin-tazobactam at 65.4%. Additive effects were also noted but to a lesser extent. The highest additive interaction occurred with piperacillin-tazobactam (34.6%), followed by doxycycline (33.3%), cefepime (30%), and imipenem (27%). Lower additive rates were observed with ciprofloxacin and gentamicin (both at 19.2%), and sulfamethoxazole-trimethoprim (2.8%), while no additive effect was detected with amikacin. Importantly, no cases of indifference (FIC >1) or antagonism (FIC >2) were detected (Figure 3), confirming that chitosan nanoemulsion either enhanced or maintained antibiotic efficacy without negative interactions.

#### Combination of antimicrobial drugs with curcumin nanoemulsion

**FIGURE 4:**
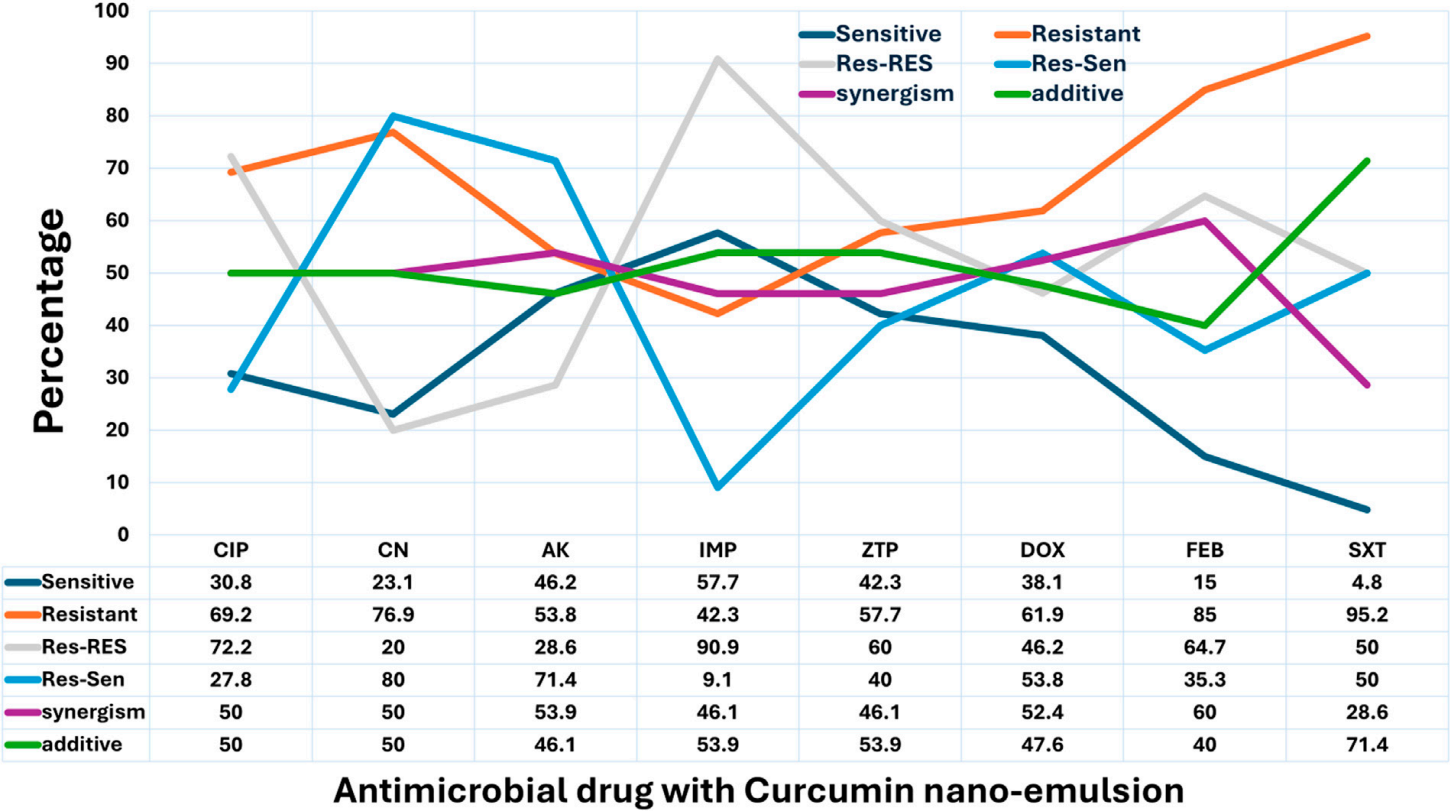
Effect of Curcumin Nanoemulsion on Antimicrobial Resistance Patterns of Pathogens in Surgical Site Infections (SSI). This line chart depicts the influence of curcumin nanoemulsion on the antimicrobial resistance patterns of various pathogens. The categories are: Sensitive (Sen), where the pathogen is susceptible; Resistant (Res), where the pathogen remains resistant; Res-Res, where initially resistant pathogens stay resistant; Res-Sen, where initially resistant pathogens become sensitive after treatment; Synergism, where the combination of curcumin nanoemulsion and the antimicrobial drug enhances its effectiveness; and Additive, where no significant change in resistance is observed. Percentages for each category reflect the effect of curcumin nanoemulsion on resistance modulation.

The resensitization potential of curcumin nanoemulsion exhibited diversity across antibiotics (Figure 4). The highest resensitization rate was observed with gentamicin (80%), followed by amikacin (71.4%), doxycycline (53.8%), and sulfamethoxazole-trimethoprim (50%). Moderate resensitization was noted with piperacillin-tazobactam (40%) and cefepime (35.3%), while lower rates were recorded for ciprofloxacin (27.8%) and imipenem (9.1%). In terms of synergistic interactions, the strongest effects were seen with cefepime (60%), amikacin (53.9%), and doxycycline (52.4%), followed by ciprofloxacin, gentamicin (both 50%), and piperacillin-tazobactam and imipenem (both 46.1%). The lowest synergism was found with sulfamethoxazole-trimethoprim (28.6%). Regarding additive effects, the highest rate was observed with sulfamethoxazole-trimethoprim (71.4%), followed by imipenem and piperacillin-tazobactam (both 53.9%), doxycycline (47.6%), and amikacin (46.1%). Ciprofloxacin and gentamicin each showed 50% additive effect, while cefepime had a slightly lower rate at 40%. No indifference (FIC >1) or antagonism (FIC >2) effects were observed in any of the combinations tested with curcumin nanoemulsion (Figure 4).

#### Combination of antimicrobial drugs with lavender nanoemulsion

**FIGURE 5:**
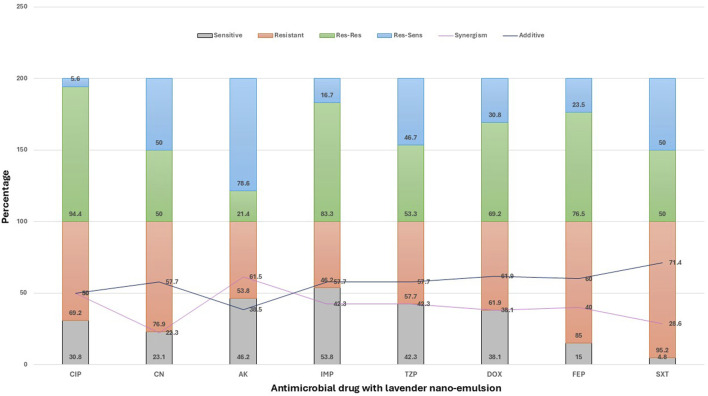
Effect of Lavender Nanoemulsion on Antimicrobial Resistance Patterns of Pathogens in Surgical Site Infections (SSI). This stacked bar chart displays the effect of lavender nanoemulsion on the antimicrobial resistance patterns of various pathogens against a range of antimicrobial agents. The results are categorized into: Sensitive (Sen), where the pathogen is susceptible; Resistant (Res), where the pathogen remains resistant; Res-Res, where initially resistant pathogens remain resistant; Res-Sen, where initially resistant pathogens become sensitive after treatment; Synergism, where the combination of lavender nanoemulsion and the antimicrobial drug enhances its effect; and Additive, indicating no significant change in resistance. Data are presented as percentages, showing the modulation of resistance patterns by lavender nanoemulsion.

The activity of lavender nanoemulsion varied across antimicrobials in terms of resensitization and interaction type (Figure 5). The highest resensitization rate was observed with amikacin (78.6%), followed by gentamicin and sulfamethoxazole-trimethoprim (both 50%), piperacillin-tazobactam (46.7%), doxycycline (30.8%), cefepime (23.5%), imipenem (16.7%), and ciprofloxacin showing the lowest rate (5.6%). Regarding synergistic interactions, the strongest effects were seen with amikacin (61.5%) and ciprofloxacin (50%). Moderate synergism was observed with imipenem and piperacillin-tazobactam (42.3% each), cefepime (40%), doxycycline (38.1%), and sulfamethoxazole-trimethoprim (28.6%). The lowest synergism was recorded with gentamicin (22.3%). In terms of additive effects, the highest rates were noted with sulfamethoxazole-trimethoprim (71.4%), followed by doxycycline (61.9%), cefepime (60%), and gentamicin, imipenem, and piperacillin-tazobactam (each 57.7%). Moderate additive interactions were found with ciprofloxacin (50%) and amikacin (38.5%). Notably, no antagonistic or indifferent effects were observed in any of the tested combinations, indicating that lavender nanoemulsion either enhanced or supported the activity of the antibiotics.

Overall, chitosan nanoemulsion showed the strongest antimicrobial efficacy, requiring the lowest concentration to inhibit all tested strains. Moreover, chitosan nanoemulsion proved to be the most effective option for restoring antibiotic potency, particularly against multidrug-resistant (MDR) pathogens, while curcumin and lavender nanoemulsions demonstrated complementary effects in antimicrobial therapy. Based on these findings, chitosan nanoemulsion was selected for further analysis.

### *In vitro* cytotoxicity testing

MTT assay results showed that chitosan nanoemulsion, the most effective formulation, maintained high cell viability (>85%) at concentrations ≤200 μg/mL over 24 and 48 h, indicating non-cytotoxicity and suitability for *in vivo* application. This concentration was thus selected for subsequent wound healing experiments. At higher concentrations (>200 μg/mL), cell viability dropped to ∼70%, suggesting potential cytotoxicity. Notably, the safe concentration range was lower than the MIC values observed for chitosan nanoemulsion alone (300–600 μg/mL). However, when combined with other antimicrobials, its MIC values dropped significantly (50–300 μg/mL), aligning more closely with the safe range. These findings suggest that while chitosan nanoemulsion alone may not suffice as a standalone antimicrobial, it shows strong potential to enhance the efficacy of co-administered agents.

### *In vivo* cytotoxicity test

In this study, *P. aeruginosa* and MRSA demonstrated high virulence, strong multidrug resistance, and robust biofilm formation, making them suitable challenge organisms for *in vivo* testing due to their prominent role in nosocomial infections. Amikacin, combined with chitosan nanoemulsion, was selected for its superior synergistic and resensitization effects. This combination offered a balanced therapeutic profile, with strong synergy, effective resensitization, and a safe MIC range (50–200 μg/mL) within non-cytotoxic limits. Additionally, amikacin’s widespread use and availability as an aminoglycoside further supported its selection for *in vivo* evaluation.

### Wound healing and scoring

#### Granulation score of wounds

**FIGURE 6:**
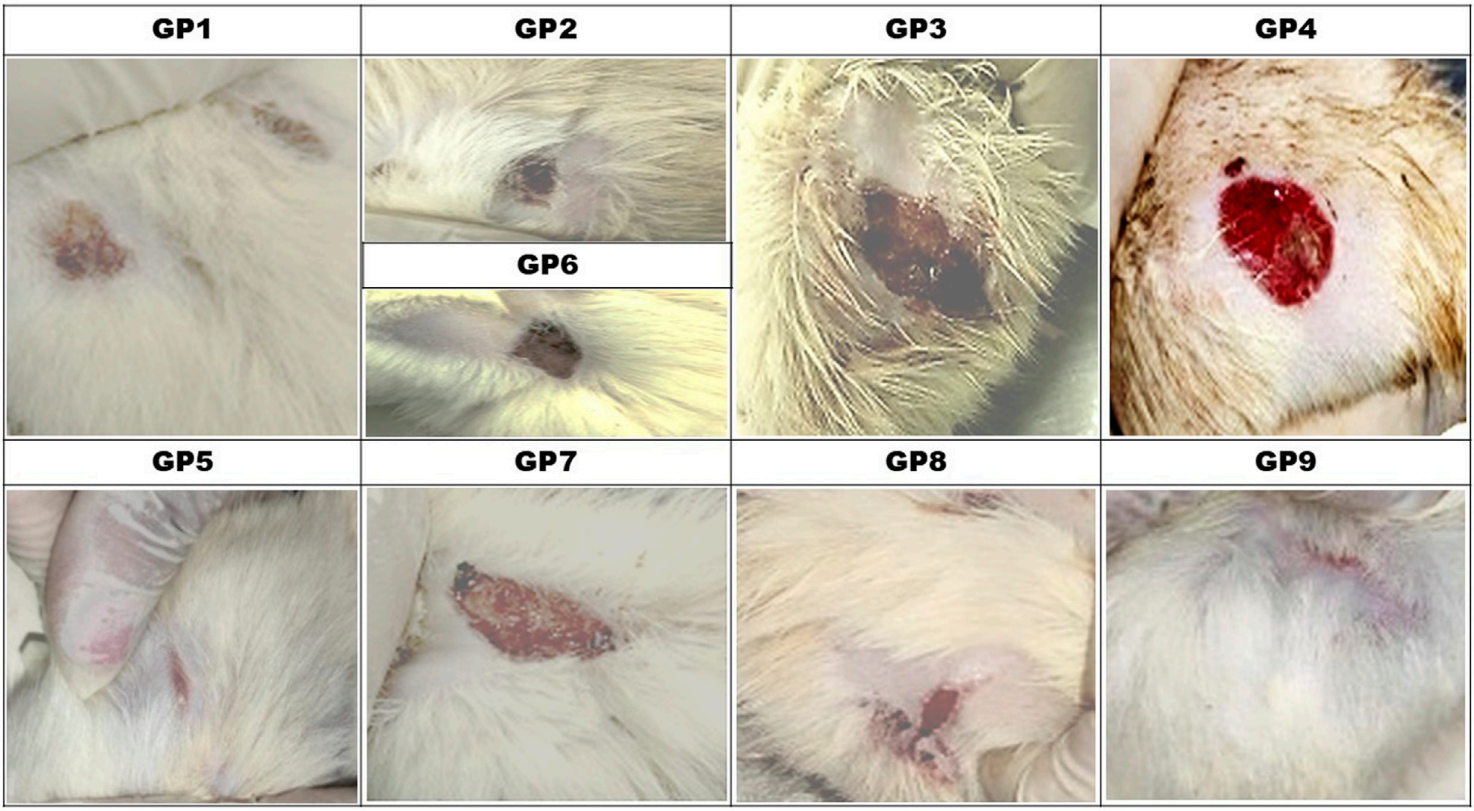
Wound Healing Score (Granulation and Inflammation) in Experimental Groups. The images demonstrate the wound healing process in animals assigned to nine experimental groups (n = 10 per group). Treatment conditions include: GP1 (non-infected, non-treated control group with placebo), GP2 (*P. aeruginosa* -infected, non-treated group), GP3 (*P. aeruginosa* -infected, treated with the most effective nanoemulsion), GP4 (*P. aeruginosa* -infected, treated with selected antimicrobial), GP5 (*P. aeruginosa* -infected, treated with a combination of antimicrobial and nanoemulsion), GP6 (*S. aureus*-infected, non-treated group), GP7 (*S. aureus* -infected, treated with the most effective nanoemulsion), GP8 (Gram-positive-infected, treated with selected antimicrobial), and GP9 (*S. aureus* -infected, treated with a combination of antimicrobial and nanoemulsion). The images display varying degrees of wound healing, with granulation and inflammation levels corresponding to the treatment protocol.

The granulation score of wounds varied among the different experimental groups, reflecting the impact of infection and treatment on wound healing (Figure 6). The non-infected, non-treated control group (G1) exhibited the highest granulation score (4), indicating optimal healing. In contrast, the non-treated infected groups with *P. aeruginosa* and MRSA (G2 and G6) showed the lowest granulation scores (1), demonstrating impaired wound healing due to bacterial infection. Treatment with chitosan nano-emulsion alone (G3 and G7) resulted in a moderate improvement, with granulation scores of 3 and 2, respectively. Administration of amikacin alone (G4 and G8) led to better healing responses, as shown by granulation scores of 2 and 3. Notably, the combination therapy groups (G5 and G9) achieved the highest granulation scores (4) among infected groups, matching the non-infected control (G1), suggesting that the combined treatment was the most effective in promoting wound healing.

#### Wound inflammation scores

Severe inflammation was observed in the untreated infected groups (G2 and G6). Both groups scored 4, indicating a strong inflammatory response associated with *P. aeruginosa* and MRSA infections, respectively (Figure 6). In contrast, the non-infected, non-treated control group (G1) exhibited the lowest score (1), reflecting minimal inflammation and normal wound healing. Treatment interventions showed varying degrees of effectiveness in reducing inflammation. Chitosan nanoemulsion-treated groups (G3 and G7) demonstrated moderate improvement, both scoring 2, suggesting its role in inflammation control. Similarly, amikacin-treated groups (G4 and G8) also recorded an inflammation score of 2, indicating a comparable anti-inflammatory effect. The most significant reduction in inflammation was observed in the combination therapy groups (G5 and G9), both achieving the lowest score (1), similar to the non-infected control. Overall, the chitosan nano-emulsion enhanced the antimicrobial activities of amikacin, provided the most significant improvement in wound granulation, reduced the inflammatory response, and enhanced recovery.

### Histopathology examination

#### Histological examination of wound healing in untreated uninfected (G1) and infected groups (G2 and G6) with *P. aeruginosa* and MRSA

**FIGURE 7:**
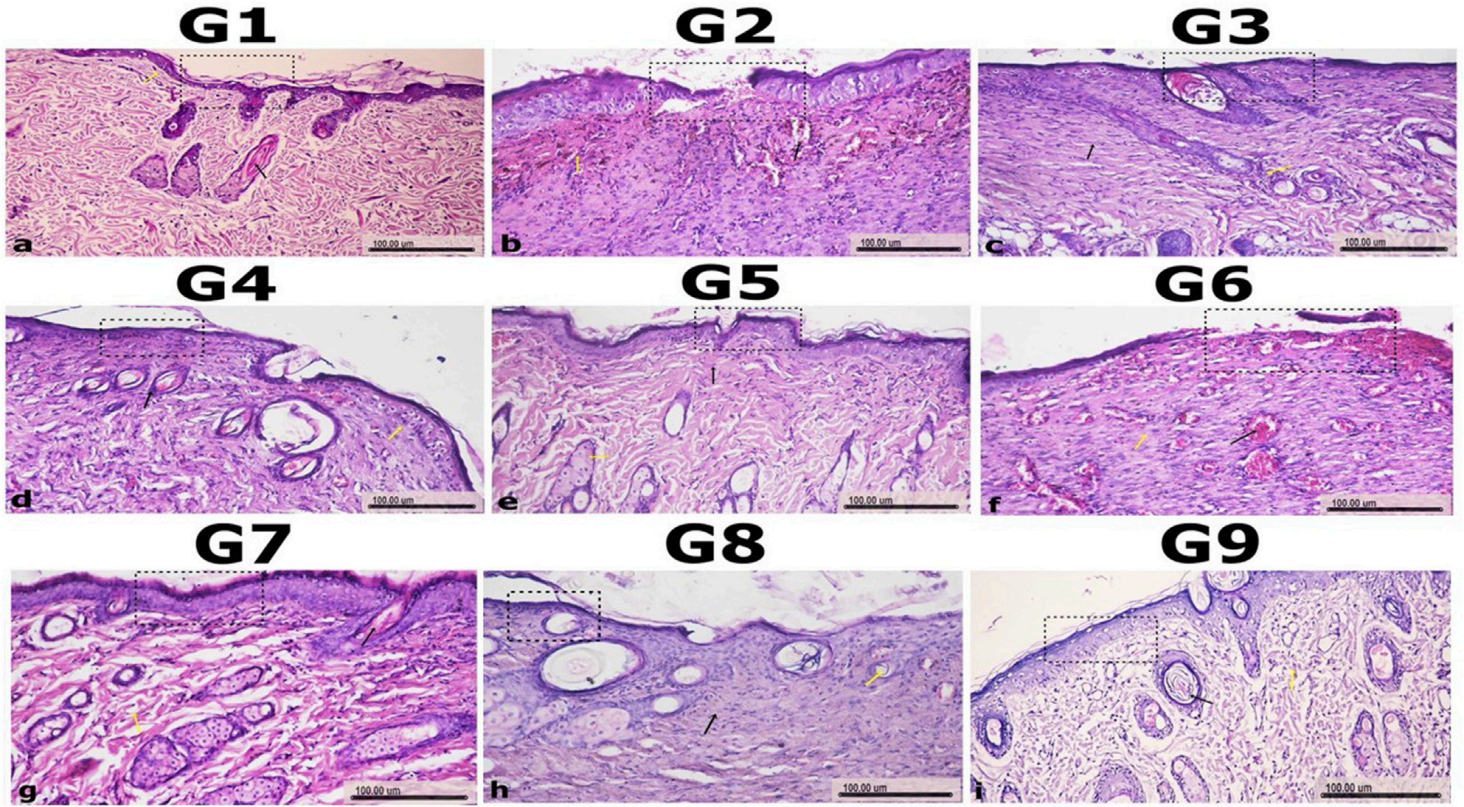
Histopathology of Wound Sections to Assess Wound Healing in Experimental Groups. The histological sections of wound tissues from nine experimental groups (G1–G9) are shown to evaluate the degree of wound healing. H&E-stained sections reveal granulation tissue, inflammatory responses, and tissue regeneration. Granulation tissue formation and inflammation are indicated by yellow and black arrows, respectively. GP1 (non-infected, non-treated control group with placebo), GP2 (*P. aeruginosa* -infected, non-treated group), GP3 (*P. aeruginosa* -infected, treated with the most effective nanoemulsion), GP4 (*P. aeruginosa* -infected, treated with selected antimicrobial), GP5 (*P. aeruginosa* -infected, treated with a combination of antimicrobial and nanoemulsion), GP6 (*S. aureus*-infected, non-treated group), GP7 (*S. aureus* -infected, treated with the most effective nanoemulsion), GP8 (Gram-positive-infected, treated with selected antimicrobial), and GP9 (*S. aureus* -infected, treated with a combination of antimicrobial and nanoemulsion) showed differences in healing, with the presence of regenerated tissue and reduced inflammation based on the treatment applied. Scale bars represent 100 µm.

The normal control group (G1) exhibited well-preserved skin architecture, with a stratified squamous keratinized epithelium in the epidermis, a thin keratin layer, and the presence of hair follicles and sebaceous glands. The dermis showed a papillary layer rich in blood vessels and fine collagen fibers, indicative of healthy skin. In contrast, the untreated infected groups (G2 and G6) displayed severe tissue damage, including sloughing and necrosis of the epidermis and superficial dermal layers. Marked inflammatory cell infiltration and hemorrhage were also observed, reflecting a severe inflammatory response associated with *P. aeruginosa* and MRSA infections (Figure 7).

#### Histological assessment of wound healing in treated groups with either amikacin or chitosan nanoemulsion

In the G3 group, moderate improvement was observed, with mild inflammation and the formation of irregular granulation tissue in the wound site, indicating the early stages of healing. The G4 group showed some recovery, with necrosis and vacuolar degeneration in some epidermal cells, alongside newly formed hair follicles, although significant tissue damage remained. The G7 group exhibited mild healing, with partial necrosis of epidermal cells and dispersion of the dermis, showing a less pronounced improvement compared to the combination-treated groups. The G8 group demonstrated moderate healing with regular granulation tissue and new hair follicles, though the recovery was still less advanced than in G9 (Figure 7).

#### Histological analysis of wound healing in groups treated with a combination of amikacin and chitosan nanoemulsion

Among the treated groups, the GP5, which received the combination treatment, demonstrated significant wound healing. Histological examination revealed re-epithelialization, with the wound area partially covered by newly formed epidermal tissue and the presence of collagen fibers filling the wound site, indicating active tissue repair. Additionally, the treated group receiving combination therapy (GP9) showed the most prominent healing response. Histological sections from this group closely resembled normal skin, featuring a thin epidermis, minimal hypertrophic scarring, and nearly normal hair growth in the wound area. These findings suggest that the combination treatment in GP5 and GP9 promoted optimal wound healing, leading to the restoration of skin integrity with minimal scarring (Figure 7).

#### Investigation of the inflammatory and anti-inflammatory biomarkers among experimental groups

**FIGURE 8:**
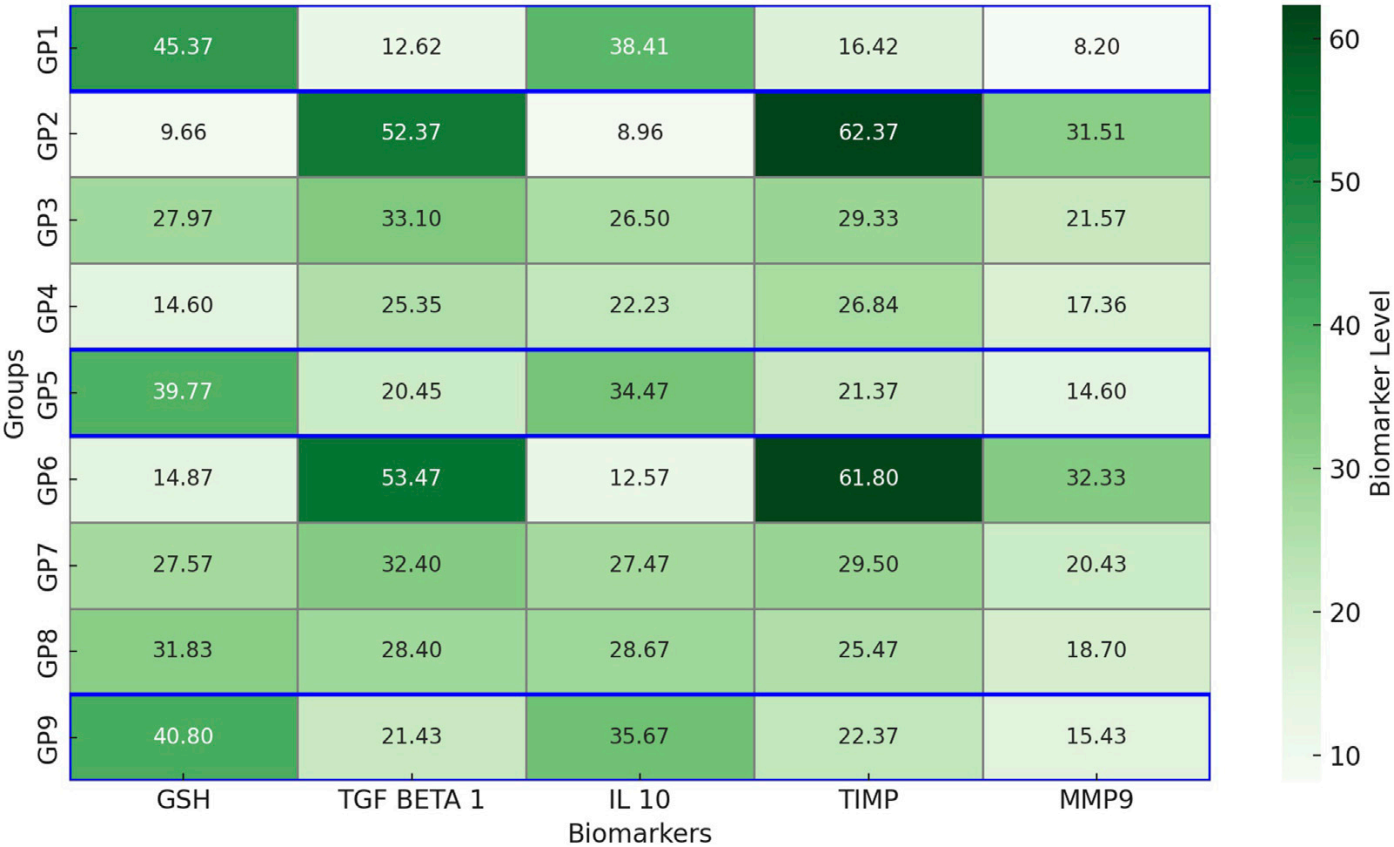
Heatmap representation of cytokine and biomarker levels across experimental groups. This heatmap illustrates the levels of five key biomarkers—glutathione (GSH), transforming growth factor beta 1 (TGF-β1), interleukin-10 (IL-10), tissue inhibitor of metalloproteinases (TIMP), and matrix metalloproteinase 9 (MMP9)—measured in nine experimental groups (GP1–GP9). Data are color-coded using a green gradient, where darker shades indicate higher concentrations. The blue markers indicate that the values for the treated infected groups receiving combination therapy (GP5 and GP9) are closest to those of the uninfected control group (GP1). TIMP, MMP9, and TGF-β1 are expressed in ng/mL, whereas GSH and IL-10 are expressed in pg/mL.

Biomarker analysis demonstrated distinct physiological responses across the experimental groups (Figure 8), reflecting the effects of infection and therapeutic intervention. GSH levels were significantly higher in the non-infected control group (GP1: 45.37 ± 0.21 pg/mL) compared to the infected, untreated group (GP2: 9.66 ± 12.95 pg/mL; *p* < 0.01), indicating pronounced oxidative stress. Combination therapy groups, GP5 (39.77 ± 0.29 pg/mL) and GP9 (40.8 ± 0.49 pg/mL), showed significantly restored GSH levels (*p* < 0.05), closely resembling control values. TGF-β1, a marker of inflammation, was significantly elevated in infected, untreated groups (GP2: 52.37 ± 0.31 ng/mL; GP6: 53.47 ± 0.33 ng/mL) compared to GP1 (12.62 ± 0.21 ng/mL; *p* < 0.01). Treatment with combination therapy significantly reduced TGF-β1 levels in GP5 (20.45 ± 0.35 ng/mL) and GP9 (21.43 ± 0.34 ng/mL; *p* < 0.05), indicating an improved inflammatory profile. IL-10, an anti-inflammatory cytokine, was highest in GP1 (38.41 ± 0.35 pg/mL) and significantly suppressed in GP2 (8.96 ± 12.40 pg/mL; *p* < 0.01). Treatment with amikacin and chitosan nanoemulsion significantly restored IL-10 levels in GP5 (34.47 ± 0.33 pg/mL) and GP9 (35.67 ± 0.45 pg/mL; *p* < 0.05), approaching control values. TIMP levels, associated with extracellular matrix regulation, were markedly elevated in GP2 (62.37 ± 0.09 ng/mL) and GP6 (61.8 ± 7.1 ng/mL) compared to GP1 (16.4 ± 16 ng/mL; *p* < 0.01), suggesting tissue remodeling imbalance. Treatment with combination therapy in GP5 (21.37 ± 0.31 ng/mL) and GP9 (22.37 ± 0.12 ng/mL) led to significant reductions (*p* < 0.05). Similarly, MMP9, a proteolytic enzyme linked to tissue degradation, peaked in GP2 (31.51 ± 0.29 ng/mL) and GP6 (32.33 ± 0.17 ng/mL), significantly exceeding GP1 levels (8.2 ± 10.04 ng/mL; *p* < 0.01). Combination treatment again proved effective, significantly lowering MMP9 in GP5 (14.6 ± 0.14 ng/mL) and GP9 (15.43 ± 0.34 ng/mL; *p* < 0.05). In summary, infection resulted in significant oxidative stress, inflammation, and tissue damage, as reflected by altered biomarker profiles. Combination therapy (GP5, GP9) effectively normalized these parameters, closely approximating those of the uninfected control group (GP1), and highlighting its therapeutic potential in managing both Gram-negative and Gram-positive bacterial infections.

#### Investigation of logarithmic bacterial count across various treatment groups for *P. aeruginosa* and MRSA infections

**FIGURE 9:**
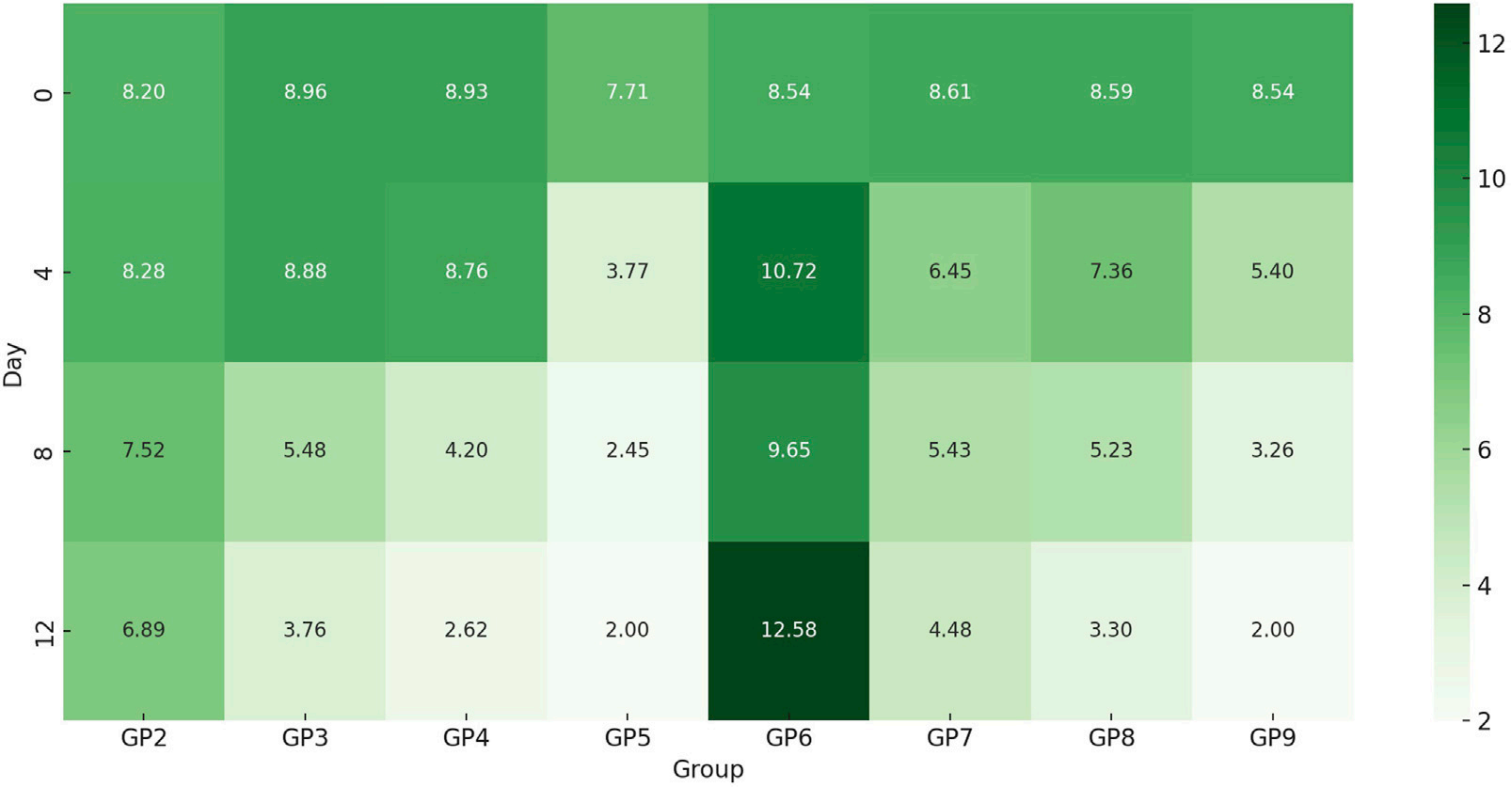
Heatmap of time-dependent changes in log bacterial burden across uninfected and infected groups with or without treatment. This heatmap illustrates the log_10_ bacterial load over time (Days 0, 4, 8, and 12) across treatment groups (GP2–GP9) infected with *P. aeruginosa* or MRSA. Color intensity represents bacterial burden, with darker green shades indicating higher bacterial counts. Untreated groups (e.g., GP2 and GP6) maintained persistently high bacterial levels, while groups receiving chitosan nanoemulsion, amikacin, or their combination showed progressive bacterial reduction.

*In vivo* analysis demonstrated variable treatment efficacies against *P. aeruginosa* and MRSA infections over a 12-day period (Figure 9). The observed differences in bacterial load reduction reflected the distinct responses of Gram-negative and Gram-positive pathogens to the applied therapies. As expected, no bacterial growth was detected in the non-infected, untreated control (GP1). In the *P. aeruginosa*-infected, untreated group, bacterial counts declined naturally (from log 8.2 to 6.89) but remained significant. Chitosan nanoemulsion alone achieved a greater reduction, indicating notable activity against Gram-negative bacteria. Amikacin treatment also reduced bacterial load, though less effectively than nanoemulsion alone. The combination of amikacin and chitosan nanoemulsion yielded the most profound effect, reducing bacterial counts to nearly undetectable levels by day 12, suggesting a strong synergistic action. In MRSA-infected groups, untreated wounds showed increased bacterial growth, while both chitosan nanoemulsion and amikacin treatments led to reduced bacterial loads—again, with greater efficacy observed for the nanoemulsion. The combination therapy proved most effective, decreasing MRSA counts from log 8.54 to below detectable limits (<100), representing the highest reduction among Gram-positive-infected groups. Statistical analysis using the Friedman test (GP2–GP9) showed a significant change in bacterial loads over time (p = 0.011), confirming time-dependent treatment effects. However, no significant differences were found between groups at individual time points, suggesting that the observed changes occurred within groups across the study period. Overall, the combination of amikacin and chitosan nanoemulsion demonstrated superior antibacterial activity against both Gram-negative and Gram-positive infections, underscoring the potential of combination therapy for enhanced treatment outcomes.

## Discussion

Surgical site infections are a major global healthcare issue, ranking among the most common healthcare-associated infections and contributing to prolonged hospitalization, higher costs, and increased morbidity and mortality (Klevens et al., 2007; Mangram et al., 1999). The WHO has identified SSIs as a significant threat to patient safety, stressing the need for effective prevention and management (World Health Organization, 2018; Keely Boyle et al., 2018). SSIs can lead to severe complications such as wound dehiscence, systemic infections, and antibiotic resistance (Calderwood et al., 2023). In this study, bacterial isolates commonly linked to SSIs including *S. aureus*, *E. coli*, *K. pneumoniae*, *P. aeruginosa*, and *A. baumannii* were detected. The resistance profiles observed in this study aligned with findings from multiple published reports. *P. aeruginosa* showed moderate-to-high sensitivity to amikacin, imipenem, and cefepime but resistance to ciprofloxacin, gentamicin, and piperacillin-tazobactam (Kindiki et al., 2025). *K. pneumoniae* exhibited low sensitivity overall, especially to piperacillin-tazobactam and doxycycline, consistent with carbapenem-resistant trends (Kopotsa et al., 2020). *S. aureus* demonstrated moderate sensitivity to ciprofloxacin and imipenem but resistance to doxycycline and piperacillin-tazobactam, aligning with rising MRSA rates (Bukharie et al., 2001). *A. baumannii* was highly resistant, with limited sensitivity to amikacin and imipenem (Ayoub-Moubareck and Hammoudi-Halat, 2020). *E. coli* was more responsive to imipenem and amikacin but resistant to piperacillin-tazobactam and doxycycline, reflecting increasing ESBL activity (Paterson and Bonomo, 2005). Therefore, these antimicrobial sensitivity patterns raised concerns about the declining efficacy of standard antibiotics. Furthermore, the detected MDR patterns, MAR index values ≥0.5, and biofilm production are concerning, as they indicate reduced efficacy of standard treatments and highlight the need for alternative therapeutic strategies and stricter infection control measures (Magiorakos et al., 2012).

The distribution of virulence genes among clinical isolates revealed a high prevalence of multivirulent strains, complicating SSI management. Notably, 50% of the MDR biofilm-producing isolates were multivirulent, enhancing bacterial pathogenicity and persistence. *P. aeruginosa* showed 41.7% multivirulent strains, while *S. aureus*, *E. coli*, *A. baumannii*, and *K. pneumoniae* ranged between 45.5% and 57.1%. These strains pose greater treatment challenges due to their ability to evade host defenses, damage tissues, and resist antimicrobials (Sendra et al., 2024). In *P. aeruginosa*, virulence genes such as *toxA*, *aprA*, and *lasB* contribute to tissue degradation and biofilm formation, undermining antibiotic efficacy (Qin et al., 2022). Likewise, *S. aureus* isolates carrying *eta* and *tst* are linked to severe outcomes like toxic shock syndrome (Lucidi et al., 2024). The strong correlation between multivirulence and biofilm production further hinders treatment, as biofilms shield bacteria from immune responses and antibiotics (Vestby et al., 2020). Combined with MDR, this limits therapeutic options and increases the risk of chronic or recurrent infections, highlighting the urgent need for more effective antimicrobials and enhanced infection control in clinical settings.

The assessment of the antimicrobial efficacy of natural nanoemulsions against multivirulent, multidrug-resistant (MDR) biofilm-producing pathogens was promising. The results showed varying degrees of antimicrobial efficacy, with chitosan nanoemulsion demonstrating the most potent activity across all pathogens involved in SSI. Chitosan nanoemulsion exhibited the lowest MIC values compared to curcumin and lavender nanoemulsions, suggesting its superior antimicrobial properties. These findings highlight the potential of chitosan-based nanoemulsions as an effective antimicrobial agent, especially in tackling multidrug-resistant biofilm-producing pathogens, which are difficult to treat with conventional antibiotics (Kravanja et al., 2019; Pacheco et al., 2024). However, the detected cytotoxic threshold (above 200 μg/mL) of the nanoemulsion limited its direct therapeutic application despite its promising antimicrobial activity (Rodrigues et al., 2012). Fortunately, when combined with conventionally used antimicrobials, the outcomes were encouraging in multiple aspects. The effective concentrations remained below the cytotoxic threshold, and strong synergistic effects were observed with imipenem, amikacin, and doxycycline, potentially enhancing antibiotic penetration or disrupting biofilms (Godoy et al., 2025). In addition, significant resensitization effects were recorded, particularly in reversing resistance among carbapenem-resistant and aminoglycoside-resistant pathogens.

The histopathological findings revealed the substantial impact of therapeutic interventions on wound healing, particularly in infections caused by multidrug-resistant (MDR) pathogens. In the absence of treatment, extensive tissue damage was observed in GP2 and GP6, illustrating the destructive effects of bacterial infections on skin structure and function (Gür et al., 2023). These observations underscored the importance of not only controlling infection but also supporting tissue repair during treatment. Chitosan nanoemulsion demonstrated notable therapeutic promise, as observed in GP4 and GP8, due to its dual action: antimicrobial activity and the ability to promote tissue regeneration. Improvements in the epidermal and dermal layers were likely attributed to its anti-inflammatory effects, stimulation of cell proliferation, and enhancement of collagen synthesis, key processes involved in tissue recovery (Rajinikanth et al., 2024; Mawazi et al., 2024).While amikacin provided some degree of infection control, its limited impact on complete tissue repair highlighted the shortcomings of antibiotic monotherapy in addressing the complex pathology of MDR infections (Mawazi et al., 2024). In contrast, the combination of chitosan and amikacin, as seen in GP5 and GP9, resulted in a markedly improved healing profile, suggesting a synergistic interaction. The enhanced outcomes observed with combination therapy can be explained by a synergistic interaction between chitosan and amikacin. Chitosan is believed to improve amikacin’s diffusion through biofilms and bacterial cell walls, increasing the local antibiotic concentration at the infection site (Godoy et al., 2025). Simultaneously, chitosan supports tissue repair by stimulating regenerative pathways. This dual action—enhancing antimicrobial efficacy while directly promoting tissue regeneration—offers a comprehensive approach to wound healing, particularly in cases complicated by persistent infections (Rajinikanth et al., 2024).

The fluctuations in inflammatory and anti-inflammatory biomarkers across groups reflected the physiological impact of infection and treatment. Infection with MDR pathogens, as seen in GP2 and GP6, induced oxidative stress and persistent inflammation, impairing tissue repair. Regulating key biomarkers was thus essential for restoring balance and promoting healing. Chitosan nanoemulsion, particularly in combination with amikacin (GP5 and GP9), contributed to biomarker normalization through several mechanisms. It increased reduced glutathione (GSH), an antioxidant that mitigates ROS-induced damage (Xia et al., 2022), supporting tissue regeneration. It also modulated TGF-β1, facilitating collagen synthesis and resolution of inflammation (Rajinikanth et al., 2024), and enhanced IL-10 levels, helping suppress excessive immune responses (Zosangpuii and Sudheer, 2024). Furthermore, chitosan downregulated MMP-9, preventing excessive matrix degradation and preserving tissue structure (Mawazi et al., 2024). These effects explained the restoration of GSH, TGF-β1, IL-10, and MMP-9 levels in treated groups, particularly in the combination therapy, which brought values closer to the non-infected control.

Regarding bacterial clearance, amikacin alone demonstrated limited effectiveness, which was likely due to common resistance mechanisms employed by multidrug-resistant (MDR) pathogens, such as biofilm formation and efflux pump activity. Biofilms, in particular, serve as physical and biochemical barriers that protect bacterial communities by restricting the penetration of antibiotics and shielding pathogens from immune responses. This phenomenon was especially evident in GP2 and GP6, where high bacterial loads persisted despite treatment. In contrast, groups treated with the combination of chitosan and amikacin (GP5 and GP9) exhibited significantly improved bacterial clearance, indicating a synergistic interaction between the two agents. Chitosan enhanced amikacin’s antimicrobial efficacy by disrupting the biofilm matrix. Through its positively charged polymeric structure, chitosan engaged in electrostatic interactions with the negatively charged components of the biofilm’s extracellular polymeric substances. This weakened the structural integrity of the biofilm and increased its permeability, thereby facilitating deeper antibiotic penetration (Godoy et al., 2025; Kravanja et al., 2019). As a result, amikacin was able to access and act on bacterial cells that were previously protected within the biofilm environment, effectively restoring its antimicrobial potency even against resistant strains. This mechanism explains the near-complete bacterial reduction observed in the combination-treated groups—an outcome that significantly surpassed the efficacy of either agent used alone. These findings underscore the therapeutic value of combination strategies in overcoming bacterial resistance and improving outcomes in the treatment of MDR infections.

## Conclusion

In conclusion, this study highlights the challenges of managing SSIs caused by MDR, multivirulent and biofilm-producing pathogens. Chitosan nanoemulsion demonstrated significant antimicrobial potential, with the lowest MIC across all tested pathogens, showing promise for enhancing conventional antibiotics’ effectiveness. The combination of chitosan nanoemulsion with antibiotics like amikacin exhibited strong synergy, reversing resistance and significantly reducing bacterial loads. This synergy was especially evident by *in vivo* wound healing experiments, which showed improved wound healing, reduced inflammation, and enhanced tissue regeneration. Overall, combination therapies using chitosan-based nanoemulsions present a valuable approach for managing SSIs caused by MDR pathogens, improving antibiotic efficacy, reducing resistance, and promoting faster healing. Further research and clinical trials are needed to optimize these therapies for real-world applications.

## Data Availability

The datasets presented in this study can be found in online repositories. The names of the repository/repositories and accession number(s) can be found in the article/Supplementary Material.

## Supplementary material

SUPPLEMENTARY FIGURE S1 Hierarchical Distribution of Detected Isolates Among Pathogens in Surgical Site Infections (SSI). The heatmap illustrates the distribution of bacterial isolates (*P. aeruginosa, S. aureus, E. coli, A. baumannii, and K. pneumoniae*) in relation to antimicrobial resistance and virulence factors. Each row represents a bacterial isolate, and columns correspond to different antimicrobial agents and resistance markers. The color intensity indicates the presence of resistance or virulence traits. Panels (A–E) correspond to *P. aeruginosa, S. aureus, E. coli, A. baumannii, and K. pneumoniae*, respectively, displaying clustering patterns of resistance profiles for various drugs and virulence factors like biofilm production and resistance genes.
