# Supplementary figures and images for: Advanced therapeutic strategy for managing surgical site infections with natural nanoemulsion-antimicrobial combination
Source: Front Pharmacol. 2025 Jul 2;16:1617184. doi: 10.3389/fphar.2025.1617184 (PMC12263955; doi:10.3389/fphar.2025.1617184)

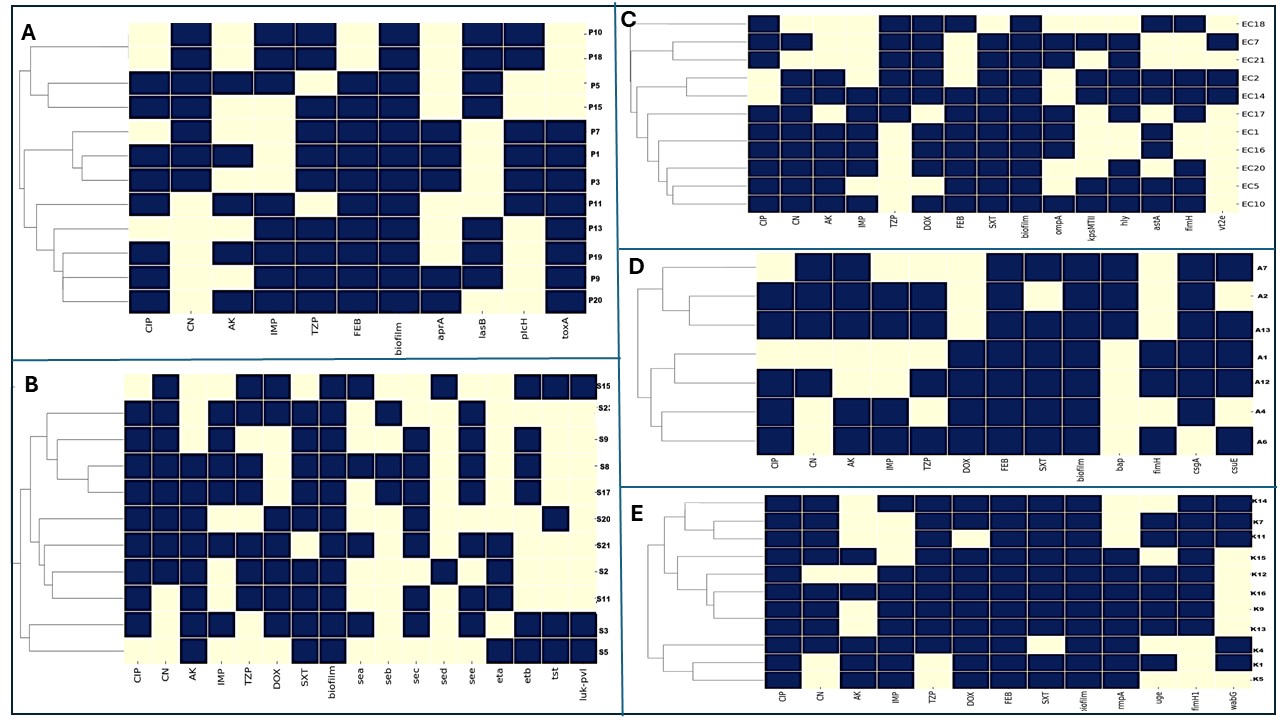

Supplement: Supplementary file 1 [file Image1.jpeg]
